# Comparison of the Effects of Three Types of Exercise (Aerobic Exercise, Resistance Training, and Mind‐Body Exercise) on Fibromyalgia: A Systematic Review and Network Meta‐Analysis of Randomized Controlled Trials

**DOI:** 10.1155/prm/1767509

**Published:** 2026-04-01

**Authors:** Yikang Pan, Kaihong Sun, Jianfeng Chen, Zhiyong Wu

**Affiliations:** ^1^ Sports Department, Changzhou Vocational Institute of Textile and Garment, Changzhou, 213164, China

**Keywords:** aerobic exercise, fibromyalgia, mind-body exercise, network meta-analysis, resistance training

## Abstract

**Background:**

Few studies have compared the effects of different types of exercise on the management of patients with fibromyalgia. Therefore, we conducted this systematic review with meta‐analysis to evaluate the effects of three types of exercise on the treatment of fibromyalgia.

**Methods:**

Search databases including PubMed, Cochrane, Embase, and Web of Science from their inception to October 15, 2022, for randomized controlled trials (RCTs) investigating the effects of exercise on people with fibromyalgia.

**Results:**

A systematic analysis was conducted on 21 RCTs involving 1638 participants and five distinct interventions. Most participants were female, consistent with the epidemiological characteristics of fibromyalgia syndrome. Compared with routine healthcare, the results of FIQ showed that more significant relief of fatigue was associated with the combination of aerobic and mind‐body exercises (SMD = −0.58 [95% CI: −1.14∼−0.01]), aerobic exercise (SMD = 0.79 [95% CI: 0.25∼1.33]), and mind‐body exercise (SMD = 0.68 [95% CI: 1.05∼−0.32]). VAS results revealed that significant improvement in pain was associated with the combination of aerobic and mind‐body exercises (SMD = −0.78 [95% CI: −1.54∼−0.02]), resistance training (SMD = −1.58 [95% CI: −2.67∼−0.49]), and mind‐body exercise (SMD = −1.62 [95% CI: −2.35∼−0.90]). Analysis of the 6MWT results showed that patients to whom the mind‐body exercise was delivered can walk farther (SMD = 33.03 [95% CI: 13.73∼52.34]), compared with those in the control group. A significant effect on 6‐minute walk test (6MWT) performance was observed following aerobic exercise (SMD = ‐23.19 [95% CI: −38.78 to −7.60]); in contrast, no significant changes were found in the group that underwent combined aerobic and resistance exercise.

**Conclusions:**

This systematic review shows the effects of single or mixed exercise intervention for the management of fibromyalgia and suggests appropriate exercise as a highly effective nonpharmacological therapeutic strategy.

## 1. Introduction

Fibromyalgia syndrome (FMS) is a chronic condition that affects approximately 2.7% of the global population, characterized by widespread pain, nonrestorative sleep, fatigue, and cognitive dysfunction [1]. The prevalence is roughly three times higher in females than in males, with the highest prevalence among individuals aged 50–60 years [2]. Moreover, FMS significantly reduces their quality of life [1, 3].

The management of FMS encompasses both pharmacologic and nonpharmacologic strategies. Pharmacologic treatments, including antidepressants and analgesics, often provide only short‐term relief and can be associated with side effects that limit long‐term use [4–6]. Consequently, current clinical guidelines prioritize nonpharmacologic interventions, with exercise therapy being the most strongly recommended component [4, 7]. Exercise therapy, as a cornerstone of FMS management, primarily includes three fundamental types: aerobic exercise, resistance training, and mind‐body exercise [8]. Aerobic exercise (e.g., brisk walking and cycling) is believed to alleviate widespread pain and fatigue by improving cardiopulmonary function and promoting the release of endogenous analgesics [2, 9]. Resistance training (e.g., using weights or bands) aims to restore muscle strength and function, thereby reducing functional limitations and potentially modulating pain perception [10, 11]. Mind‐body exercises (e.g., yoga and Tai Chi) focus on the integration of physical movement with mental focus and breath control, which may improve symptoms by reducing distress, enhancing body awareness, and improving self‐efficacy in pain management [12–14].

Therefore, we conducted this Bayesian network meta‐analysis to systematically assess and compare the effects of three fundamental exercise types (aerobic, resistance, and mind‐body) and their combinations in patients with FMS. This study aims to quantify the comparative effectiveness of these intervention strategies on key FMS symptoms, including pain, fatigue, and physical function, thereby providing robust evidence to inform clinical decision‐making for personalized exercise prescription.

## 2. Methods

The present systematic review was registered in the PROSPERO database (ID: CRD42022352859) and followed the Preferred Reporting Items for Systematic Reviews and Meta‐Analyses for Network Meta‐Analyses (PRISMA‐NMA) [15] and the Cochrane Intervention Review (Appendix 1).

### 2.1. Definitions of Exercise Interventions

Aerobic exercise: activities elevating heart rate to 64%–76% maximum (moderate intensity) or 77%–95% (vigorous intensity), such as brisk walking or cycling [16, 17].

Mind‐body exercise: interventions integrating physical postures, breath control, and mindfulness (e.g., yoga and Tai Chi) [14, 18].

Resistance training∗∗: exercises using external resistance (e.g., weights and bands) at 60%–80% of 1RM, performed 2‐3 times weekly [19, 20].

In addition to these three predefined exercise types, our network meta‐analysis also incorporated and evaluated intervention arms from the included RCTs that implemented combinations of these fundamental types (specifically, aerobic exercise combined with mind‐body exercise and aerobic exercise combined with resistance training).

### 2.2. Inclusion Criteria

Papers meeting the following criteria were included:

1. *Population (P)*: adult patients (≥ 18 years) diagnosed with FMS according to internationally recognized criteria (e.g., the American College of Rheumatology 1990, 2010, or 2016 revised criteria)
2. *Intervention (I)*: structured exercise programs, including aerobic exercise, resistance training, mind‐body exercise, or any combination thereof.
3. *Comparison (C)*: routine healthcare (e.g., general lifestyle advice and basic symptom management) that did not involve a structured exercise program.
4. *Outcomes (O)*: at least one of the following primary outcomes: fibromyalgia impact questionnaire (FIQ), visual analog scale (VAS) for pain, or the six‐minute walk test (6MWT).
5. *Study design (S)*: randomized controlled trials (RCTs).

### 2.3. Exclusion Criteria

Studies were excluded for the following reasons:

1. Non‐RCT designs (e.g., reviews, animal studies, conference abstracts, and protocols);
2. Exercise interventions that were combined with other active nonpharmacological therapies (e.g., cognitive behavioral therapy and acupuncture) unless the same adjunct therapy was also applied to the control group;
3. Participants received specific therapeutic medications initiated or adjusted for fibromyalgia pain (e.g., antidepressants, anticonvulsants, and analgesics) throughout the study period [21, 22].
4. Incomplete or insufficient data for analysis.
5. Unpublished dissertations and conference abstracts.

### 2.4. Literature Search

PubMed, Cochrane, Web of Science, and Embase were searched until October 15, 2022. Subjective terms and keywords were used for the search. The detailed search strategy is presented in Table S1.

### 2.5. Screening Literature and Extracting Data

Articles retrieved from the databases were imported to the software Endnote X9 to remove duplicates by scanning the titles and abstracts. Then, the full text was read to screen eligible articles.

Before data extraction, a standard data extraction spreadsheet was designed. The extracted information included title, author, gender, age, country, study design, course of the disease, intervention, intervention time, follow‐up time, and outcome indicators. Two researchers undertook the literature screening and data extraction independently. Any disagreements were tackled by discussion with a third researcher.

### 2.6. Assessing Study Quality

RCT risk of bias assessment tool from Cochrane Collaboration [23] was utilized by two researchers to assess the quality of included studies considering the following seven domains: generation of random sequence, allocation concealment, blinding of participants and personnel, blinding of evaluators, incomplete outcomes, selective reporting, and other bias sources. The quality of studies was rated as low risk, high risk, or unclear risk of bias.

### 2.7. Outcome Measures

#### 2.7.1. FIQ

The FIQ assesses the symptoms and dysfunction of FMS patients through a series of questions. The questionnaire contains 10 items covering the patient’s physical functioning, work status, symptoms (e.g., pain, fatigue, stiffness, anxiety, and depression), and overall health status. Each item is scored on a scale ranging from 0 to 10, with higher scores indicating more severe symptoms. Patients complete the questionnaire based on their condition over the past week. The researcher adds the item scores to arrive at a total score, which is used to assess the impact of fibromyalgia on the patient [24].

#### 2.7.2. VAS

The VAS is a simple pain assessment tool that provides quick access to the pain intensity, is easy to administer, and is easy for patients to understand and use. It takes the form of a 10 cm‐long straight line, with the left end indicating “no pain” and the right end indicating “most severe pain.” The patients mark the pain intensity they feel on the line, and the researcher measures the distance from the marked point to the left end and records the score in centimeters. Scores range from 0 to 10, with higher scores indicating more severe pain. The method is simple and intuitive and facilitates a quick assessment of the patient’s pain level [25].

#### 2.7.3. 6MWT

The 6MWT is a standardized, submaximal exercise test used to assess functional exercise capacity in patients with chronic conditions, including FMS 26. It measures the maximum distance an individual can walk on a flat, hard surface in 6 minutes. The distance walked (in meters) provides a composite measure reflecting the integrated responses of the cardiorespiratory, circulatory, neuromuscular, and metabolic systems during submaximal exertion [26]. Therefore, it is considered a reliable measure of overall functional status and the ability to perform daily physical activities, rather than a direct measure of isolated physiological functions, such as peak cardiorespiratory endurance or localized muscular endurance [27].

### 2.8. Statistical Analysis

The effectiveness of multiple interventions was assessed through Bayesian analysis of random‐effect models. The Markov chain Monte Carlo (MCMC) method was utilized for modeling with 4 chains. Models were constructed through 50,000 irritations with annealing being 20,000. The model fitting and consistency were evaluated using the deviation information standard (DIC). For the presence of a closed loop in the network, the local consistency was analyzed using the node splitting analysis. In addition, the intervention measures were ranked based on SUCRA, and league tables were generated to compare the effectiveness of various interventions. Funnel plots were drawn to evaluate the heterogeneity across studies. Data analysis was carried out using Stata 15.0 (Stata Corporation, College Station, TX) and R (Version 4.2.0, from R Development Core Team, Vienna). *p* < 0.05 indicated a significant difference.

## 3. Results

### 3.1. Literature Screening

**FIGURE 1:**
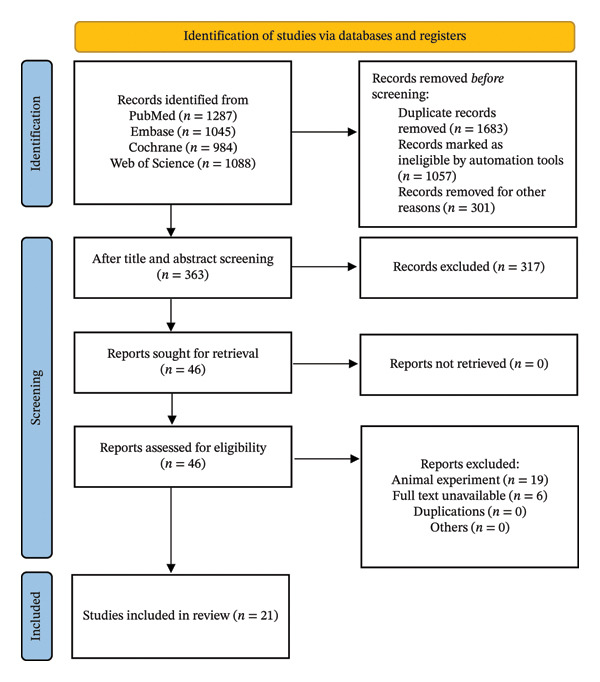
The flow diagram of study selection.

A total of 4404 studies were retrieved from databases, including 1683 duplicates, 1057 documents marked as unqualified by automated tools, and 301 documents excluded for other reasons. A total of 363 articles were obtained after reading the title and abstract, and 46 were identified after full‐text assessment. Among the remaining 46 articles, 19 were animal experiments, and 6 articles were irrelevant to the topic. Finally, 21 studies were included for meta‐analysis (Figure 1). The literature search strategy is displayed in Table S1.

### 3.2. Study Characteristics

**FIGURE 2:**
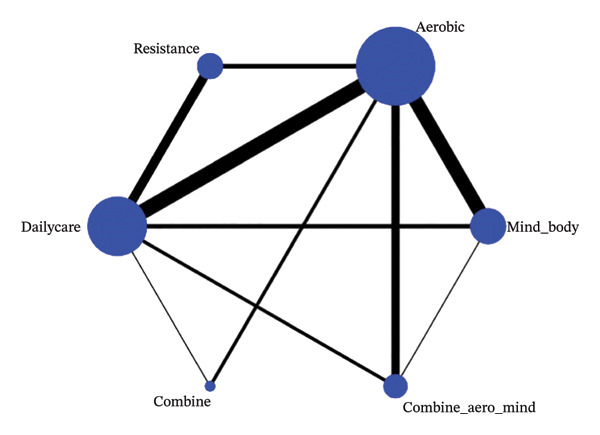
FIQ network graph.

**FIGURE 3:**
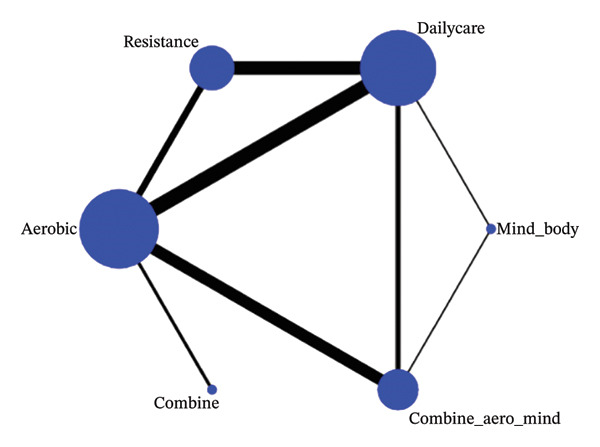
VAS network graph.

**FIGURE 4:**
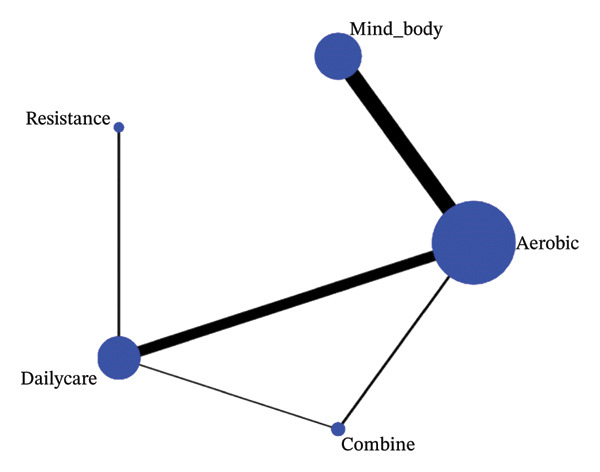
6MWT network graph.

A total of 21 RCTs involving 1638 participants with an average age of 49.63 were eligible for meta‐analysis. Among them, six studies investigated three groups (one group with routine healthcare and two groups with different exercise interventions). Fifteen studies investigated two groups (one control group and one exercise intervention group). All study participants were not diagnosed with diseases other than FMS. Exercise programs implemented in 21 RCTs included exergames from VirtualEx‐FM [10, 28], walk [29–32], light aerobic exercise [26, 30, 33–39], bicycling [37, 40], dance [41], muscle strength exercise [38, 42], Tai Chi [29], and Qigong [43, 44]. Three studies [26, 30, 45] evaluated the synergistic effect of aerobic, flexibility, and stretching exercises. In the control group, aerobic exercise was used in four studies [26, 29, 31], relaxation therapy in three studies [35, 38, 42], and daily activities in the rest of the studies (Figures 2, 3, and 4).

### 3.3. Risk of Bias

Two reviewers evaluated the quality of 21 RCTs using the Cochrane risk‐of‐bias tool (Figures S1 and S2). The results showed that 10 RCTs [29, 31–33, 37, 38, 41–44] mentioned allocation concealment, 3 RCTs [30, 31, 44] did not achieve blinding, 3 RCTs [34, 35, 45] did not describe blinding, 2 RCTs [37, 46] used double‐blinding, and the rest of the studies employed single‐blinding. There was a high rate of loss to follow‐up in 6 RCTs [10, 29, 32–34, 43].

### 3.4. Meta‐Analysis Results

#### 3.4.1. FIQ

In light of the relief of fatigue, the combination of aerobic and mind‐body exercises effectively relieved fatigue in FMS patients compared with routine care (−0.58, 95% CI: −1.14 ∼ −0.01). Aerobic exercise alone was more effective than the combination of aerobic and resistance exercises (0.62, 95% CI: 0.08–1.16), while resistance training was inferior to mind‐body and aerobic exercises (0.63, 95% CI: 0.14–1.12; 0.39, 95% CI: 0.09–0.69). Mind‐body exercise and aerobic exercise exerted favorable antifatigue effects on FMS patients compared with routine care (−0.68, 95% CI: −1.05 ∼ −0.32; 0.79, 95% CI: 0.25–1.33) (Table S2 and Figure S3). No publication bias was observed in the funnel plot (Figure S4).

#### 3.4.2. VAS

As shown in Table S3 and Figure S5, different exercise interventions relieved the pain in FMS patients to different degrees. Compared with routine care, the combination of aerobic and mind‐body exercises, resistance training alone, and mind‐body exercise alone significantly relieved pain (−0.78, 95% CI: −1.54 ∼ −0.02; −1.58, 95% CI: −2.67 ∼ −0.49; −1.62, 95% CI: −2.35 ∼ −0.90). The combination of aerobic and resistance exercises had no better effects on pain relief (1.18, 95% CI: 0.24–2.11). Resistance training significantly relieved pain compared to routine care (−0.85; 95% CI: −1.52 ∼ −0.17), and the most significant program in pain relief was resistance training (1.22, 95% CI: 0.77–1.68). Additionally, mind‐body exercise also relieved pain compared with aerobic exercise (−1.38; 95% CI; −2.12 ∼ −0.64). No significant publication bias was found according to the funnel plot (Figure S6).

#### 3.4.3. 6MWT

FMS patients who did mind‐body exercise walked further in the 6MWT compared with those receiving routine care (33.03, 95% CI: 13.73–52.34). Aerobic exercise also had positive effects on patients in the 6MWT (−23.19, 95% CI: −38.78 ∼ −7.60) (Table S4 and Figure S7). No significant publication bias was found according to the funnel plot (Figure S8).

## 4. Discussion

This study is the first to comprehensively compare the multifaceted effects (specifically on pain, fatigue, and functional status) of aerobic exercise, resistance training, and mind‐body exercise on FMS patients using systematic review and network meta‐analysis methods. Our analysis covered multiple symptom dimensions using the FIQ, VAS, and 6MWT as assessment metrics and explored in detail the differential effects of the interventions on specific symptoms (e.g., fatigue and pain). Our analysis covered multiple symptom dimensions such as pain, fatigue, and functional status, using the FIQ, VAS, and 6MWT as assessment metrics, and explored in detail the differential effects of the interventions on specific symptoms (e.g., fatigue and pain). For example, aerobic and mind‐body exercises have shown better therapeutic effects on improving FMS and physical function, and the combination of the two exercises produces the best therapeutic effects. Mind‐body exercises (e.g., Tai Chi and Qigong), which have often been overlooked in previous studies, play an important role in the management of FMS. These findings provide new ideas for developing more effective treatment plans in the future and provide important references for individualized exercise prescriptions.

### 4.1. Consistency and Generalizability of Findings

The efficacy of exercise interventions in FMS management is further supported by a growing body of evidence. Regarding fatigue reduction (FIQ), our findings align with Bidonde et al.’s Cochrane review (*n* = 839), which demonstrated the benefits of aerobic exercise on health‐related quality of life and fatigue [47]. Notably, combined interventions may offer superior effects. Kang et al. (*n* = 120) reported an 18% greater FIQ improvement in groups combining aerobic and mind‐body exercises compared to conventional care [48]. This is further supported in mind‐body exercise trials. Wang et al. (*n* = 8 RCTs) found Tai Chi significantly improved fatigue, sleep quality, and depression [49], while Victorson et al.’s systematic review highlighted sustained benefits of Qigong on pain thresholds and central sensitization [50]. For pain relief (VAS), our results resonate with Geneen et al.’s analysis (*n* = 1381), confirming exercise’s role in alleviating chronic pain [9]. Recent RCTs provide granular insights. Smith observed a 35% VAS reduction (6.5 ⟶ 4.2) and 27% FIQ decline (75 ⟶ 55) following 12‐week aerobic exercise [51], while Lee (*n* = 98) demonstrated a 50% pain reduction in combined intervention groups [52]. These findings collectively underscore the dual impact of aerobic exercise on pain and functional capacity.

Regarding functional capacity (6MWT), our results corroborate Villafaña et al.’s systematic review (*n* = 322), where exercise significantly increased walking distances [10]. Rodríguez‐Almagro further quantified these benefits and showed that aerobic interventions improved 6MWT distances by 33 m compared to controls [53]. Notably, resistance training showed divergent effects: while improving muscle strength [54], its limited impact on fatigue aligns with our findings [54, 55]. This emphasizes the need for multimodal approaches targeting both peripheral strength and central fatigue pathways.

### 4.2. Mechanisms of Action and Synergistic Effects

The differential and synergistic effects observed in our analysis can be interpreted through the distinct physiological and psychological mechanisms targeted by each exercise modality. Aerobic exercise is posited to primarily confer its benefits through peripheral adaptations. These include improvements in mitochondrial biogenesis and function in skeletal muscles, enhanced endothelial function, and a reduction in systemic oxidative stress and inflammation, which collectively may alleviate pain and fatigue by addressing underlying metabolic dysregulation and improving tissue oxygenation [47, 56]. In contrast, mind‐body exercises (e.g., yoga and Tai Chi) are thought to exert their influence predominantly on central nervous system pathways. Regular practice has been shown to modulate the activity of the hypothalamic‐pituitary‐adrenal (HPA) axis, reduce central sensitization, and enhance functional connectivity within brain regions involved in pain perception and emotional regulation, such as the insula and prefrontal cortex [57–59]. Resistance training, while effective in restoring muscle mass and strength (1RM↑30%) and thereby improving functional capacity, appears to have a more limited direct impact on central fatigue pathways, which may explain its comparatively smaller effect on fatigue scores in our analysis [60].

The superior efficacy of combining aerobic and mind‐body exercises suggests a synergistic interaction between these peripheral and central mechanisms. By simultaneously improving peripheral tissue health and modulating central pain and stress processing, a combined intervention may provide a more comprehensive attack on the multifaceted pathophysiology of FMS than any single modality alone [48]. This mechanistic framework strongly supports current clinical guidelines that advocate for multimodal, individualized exercise programs as a cornerstone of FMS management [4].

### 4.3. Considerations for Exercise Prescription and Individualization

While our findings provide strong evidence for the efficacy of different exercise modalities, a formal dose‐response relationship regarding optimal exercise intensity, frequency, or duration could not be established from the available literature, as most primary studies did not systematically compare different doses. Future studies are needed to address this important gap. Nonetheless, our results offer crucial insights for individualizing exercise prescription based on the predominant symptoms and characteristics of the patient.

The finding that mind‐body exercises were particularly effective for pain relief suggests that for patients with severe pain or high pain sensitivity, initiating treatment with these lower‐intensity, mindfulness‐based modalities may enhance tolerability and adherence [59]. In contrast, aerobic exercise yielded significant benefits for fatigue and physical function. However, its intensity must be carefully calibrated, as initially excessive intensity may exacerbate symptoms in some individuals [55]. Therefore, a stratified and progressive approach is recommended. For instance, a patient with predominant pain could begin with a foundation of mind‐body exercise to establish movement confidence and reduce pain, after which aerobic exercise can be gradually incorporated at a low‐to‐moderate intensity and slowly progressed as tolerance allows [55].

The superior efficacy of combining aerobic and mind‐body exercises underscores that the optimal “prescription” is likely not a single type of exercise, but a personalized combination. Clinicians should consider the patient’s symptom profile, preferences, and functional level when designing such multimodal programs, aligning with the principles of personalized medicine in chronic pain management [61].

### 4.4. Long‐Term Effects and Adherence

Although our meta‐analysis demonstrated the short‐term benefits of various exercise modalities, the chronic nature of FMS requires investigation of long‐term effects and strategies to maintain patient compliance. The superior efficacy of the combination interventions observed in our study may be attributed in part to improved adherence, as a variety of exercise types can help maintain patient interest and motivation. However, adherence to exercise programs remains a significant challenge in the management of FMS. Factors such as pain exacerbation, fatigue, and psychological barriers may hinder long‐term participation in exercise programs in FMS patients [62]. Future research should explore strategies to improve adherence, such as gradual exercise progression, cognitive‐behavioral interventions, and technology‐based support systems. The importance of long‐term adherence was demonstrated in a longitudinal study by Sañudo et al., showing that only those FMS patients who continue to exercise regularly can maintain the benefits of exercise [62].

In conclusion, this network meta‐analysis provides strong evidence to support the combined aerobic and mind‐body exercises for the management of FMS. The differential effects of different exercise modalities on fatigue, pain, and function underscore the need for individualized, multimodal exercise prescriptions. By gaining a deeper understanding of the effectiveness and mechanisms of exercise in FMS, we can continue to improve the management of this challenging disease and enhance the quality of life of FMS patients.

### 4.5. Limitations Section

#### 4.5.1. Sample Heterogeneity

As the included studies came from different regions and cultural backgrounds, there may be differences in patient characteristics and treatment protocols, leading to heterogeneity of results. This heterogeneity may affect our overall understanding of the effects of different exercise interventions.

#### 4.5.2. Differences in Duration and Frequency of Interventions

The duration and frequency of exercise interventions varied across studies, which may pose a challenge for comparison and comprehensive analysis of results. Future studies should standardize intervention protocols to ensure comparable results.

#### 4.5.3. Limitations of Follow‐Up Duration

Most included studies had a short follow‐up duration to adequately assess the effects of long‐term exercise interventions. Extending the follow‐up time to observe long‐term efficacy and maintain effects will be the focus of future studies.

#### 4.5.4. Lack of Outcome Indicators

FIQ, VAS, and 6MWT can only partially reflect the symptoms and functional status of FMS patients. Future studies may consider incorporating additional outcome measures, such as fatigue severity, sleep quality, depression, and cognitive function, to more fully assess the effects of exercise interventions.

#### 4.5.5. Literature Search Date

Our literature search was conducted up to October 15, 2022. Therefore, any high‐quality RCTs published after this date are not included in the current analysis.

## 5. Conclusion

In summary, this systematic review and network meta‐analysis comprehensively compares the efficacy of different exercise interventions for FMS. By integrating data from 21 RCTs with a total of 1638 patients and using multiple outcome measures (FIQ, VAS, and 6MWT), this study offers high‐level evidence for the beneficial effects of aerobic exercise, mind‐body exercise, and their combination in alleviating symptoms and improving physical function in FMS patients. Notably, the findings suggest that a combination of aerobic and mind‐body exercises may offer the most effective therapeutic advantages, providing important guidance for tailoring exercise prescriptions in clinical practice. This study lays a solid foundation for future research to refine and expand upon these findings, ultimately improving clinical decision‐making and patient outcomes in FMS management.

**Nomenclature**
FMS: Fibromyalgia syndrome
RCTs: Randomized controlled trials
FIQ: Fibromyalgia impact questionnaire
VAS: Visual analogue scale
6MWT: Six‐minute walk test
VAS: Visual analog scale
MCMC: Markov chain Monte Carlo
DIC: Deviation information standard

## Author Contributions

Yikang Pan contributed to the conceptualization, methodology, validation, formal analysis, investigation, and data curation and wrote the original draft of the manuscript. Yikang Pan also participated in the review and editing process. Kaihong Sun was involved in the methodology, software implementation, formal analysis, investigation, data curation, and visualization of the research. Jianfeng Chen contributed to the methodology, validation, and investigation. Jianfeng Chen also participated in the review and editing of the manuscript. Zhiyong Wu, as the corresponding author, contributed to the conceptualization and validation of the study. Zhiyong Wu was responsible for supervision and project administration. Zhiyong Wu also played a crucial role in the review and editing of the manuscript and provided critical feedback and guidance throughout the research process.

## Funding

No funding was received for this research.

## Disclosure

All authors read and approved the final manuscript.

## Ethics Statement

The studies included in this meta‐analysis were in accordance with the 1964 Helsinki Declaration and its later amendments or comparable ethical standards. The study did not involve humans or animals. Ethics approval is not applicable.

## Consent

The authors have nothing to report.

## Conflicts of Interest

The authors declare no conflicts of interest.

## Supporting Information

Supporting figures:

Figure S1: Risk of bias graph (percentage form).

Figure S2: Risk of bias graph.

Figure S3: Meta‐analysis of FIQ.

Figure S4: Funnel plot of FIQ.

Figure S5: Meta‐analysis of VAS.

Figure S6: Funnel plot of VAS.

Figure S7: Meta‐analysis of 6MWT.

Figure S8: Funnel plot of 6MWT.

Supporting tables:

Table S1: Literature search strategy.

Table S2: League table regarding FIQ.

Table S3: League table regarding VAS.

Table S4: League table regarding 6MWT.

Appendix 1: PRISMA‐NMA checklist.

## Supporting information


**Supporting Information** Additional supporting information can be found online in the Supporting Information section.

## Data Availability

The datasets used and/or analyzed during the current study are available from the corresponding author on reasonable request.
