# Supplementary material for: Comparison of the Effects of Three Types of Exercise (Aerobic Exercise, Resistance Training, and Mind‐Body Exercise) on Fibromyalgia: A Systematic Review and Network Meta‐Analysis of Randomized Controlled Trials
Source: Pain Res Manag. 2026 Apr 1;2026:1767509. doi: 10.1155/prm/1767509 (PMC13042347; doi:10.1155/prm/1767509)
Supplement: Supplementary file 1 — Supporting Information Additional supporting information can be found online in the Supporting Information section. [file PRM-2026-1767509-s001.zip › Supplementary Materials.docx]

# Supplementary Materials

**Supplementary Tables**

Supplementary Table 1. Literature search strategy

Supplementary Table 2. League table regarding FIQ

Supplementary Table 3. League table regarding VAS

Supplementary Table 4. League table regarding 6MWT

**Supplementary Figures**

Supplementary Figure 1. Risk of bias graph (Percentage form)

Supplementary Figure 2. Risk of bias graph

Supplementary Figure 3. Meta-analysis of FIQ

Supplementary Figure 4. Funnel plot of FIQ

Supplementary Figure 5. Meta-analysis of VAS

Supplementary Figure 6. Funnel plot of VAS

Supplementary Figure 7. Meta-analysis of 6MWT

Supplementary Figure 8. Funnel plot of 6MWT

**Supplementary Table 1** Literature search strategy

**1.Pubmed**

| Search number | Query |
| --- | --- |
| #1 | "**Fibromyalgia**"[Mesh] |
| #2 | **((((((((((((((((fibromyalgia[Title/Abstract]) OR (Fibromyalgias[Title/Abstract])) OR (Fibromyalgia-Fibromyositis Syndrome[Title/Abstract])) OR (Fibromyalgia Fibromyositis Syndrome[Title/Abstract])) OR (Syndrome, Fibromyalgia-Fibromyositis[Title/Abstract])) OR (Rheumatism, Muscular[Title/Abstract])) OR (Muscular Rheumatism[Title/Abstract])) OR (Fibrositis[Title/Abstract])) OR (Fibrositides[Title/Abstract])) OR (Myofascial Pain Syndrome, Diffuse[Title/Abstract])) OR (Diffuse Myofascial Pain Syndrome[Title/Abstract])) OR (Fibromyositis-Fibromyalgia Syndrome[Title/Abstract])) OR (Syndrome, Fibromyositis-Fibromyalgia[Title/Abstract])) OR (Fibromyalgia, Secondary[Title/Abstract])) OR (Fibromyalgias, Secondary[Title/Abstract])) OR (Secondary Fibromyalgia[Title/Abstract])) OR (Fibromyalgia, Primary[Title/Abstract])** |
| #3 | #1 OR #2 |
| #4 | "**Exercise**"[Mesh] |
| #5 | **((((((((((((((((Exercise[Title/Abstract]) OR (Exercises[Title/Abstract])) OR (Physical Activity[Title/Abstract])) OR (Activities, Physical[Title/Abstract])) OR (Activity, Physical[Title/Abstract])) OR (Physical Activities[Title/Abstract])) OR (Walking[Title/Abstract])) OR (Jogging[Title/Abstract])) OR (Running[Title/Abstract])) OR (swimming[Title/Abstract])) OR (cycling[Title/Abstract])) OR (Exercise, Physical[Title/Abstract])) OR (Exercises, Physical[Title/Abstract])) OR (Physical Exercise[Title/Abstract])) OR (Acute Exercise[Title/Abstract])) OR (Exercise, Acute[Title/Abstract])) OR (Exercise, Isometric[Title/Abstract])** |
| #6 | #4 OR #5 |
| #7 | **Resistance Training**[Mesh] |
| #8 | **(((((((((((((((((Resistance Training[Title/Abstract]) OR (Training, Resistance[Title/Abstract])) OR (Strength Training[Title/Abstract])) OR (Training, Strength[Title/Abstract])) OR (Weight-Lifting Strengthening Program[Title/Abstract])) OR (Strengthening Program, Weight-Lifting[Title/Abstract])) OR (Strengthening Programs, Weight-Lifting[Title/Abstract])) OR (Weight-Lifting Exercise Program[Title/Abstract])) OR (Exercise Programs, Weight-Lifting[Title/Abstract])) OR (Weight-Bearing Strengthening Program[Title/Abstract])) OR (Strengthening Programs, Weight-Bearing[Title/Abstract])) OR (Weight-Bearing Strengthening Programs[Title/Abstract])) OR (Weight-Bearing Exercise Program[Title/Abstract])) OR (Exercise Program, Weight-Bearing[Title/Abstract])) OR (free weights[Title/Abstract])) OR (weight machines[Title/Abstract])) OR (elastic bands[Title/Abstract])) OR (body weight exercises[Title/Abstract])** |
| #9 | #7 OR #8 |
| #10 | **((((((((((((Mind-body exercise[Title/Abstract]) OR (physical[Title/Abstract] AND cognitive training[Title/Abstract])) OR (meditation[Title/Abstract])) OR (Relaxations[Title/Abstract])) OR (progressive relaxation[Title/Abstract])) OR (flexibility training[Title/Abstract])) OR (balance training[Title/Abstract])) OR (yoga[Title/Abstract])) OR (Tai ji[Title/Abstract])) OR (tai chi[Title/Abstract])) OR (Taijiquan[Title/Abstract])) OR (Qigong[Title/Abstract])) OR (Ch'i Kung[Title/Abstract])** |
| #11 | #9 OR #10 |
| #12 | #3 AND #11 |

**2.Cochrane**

| Search number | Query |
| --- | --- |
| #1 | MeSH descriptor: [Fibromyalgia] explode all trees |
| #2 | (Fibromyalgia):ti,ab,kw OR (Fibromyalgias):ti,ab,kw OR (Fibromyalgia-Fibromyositis Syndrome):ti,ab,kw OR (Fibromyalgia Fibromyositis Syndrome):ti,ab,kw OR (Fibromyalgia-Fibromyositis Syndromes):ti,ab,kw |
| #3 | (Syndrome, Fibromyalgia-Fibromyositis):ti,ab,kw OR (Syndromes, Fibromyalgia-Fibromyositis):ti,ab,kw OR (Rheumatism, Muscular):ti,ab,kw OR (Muscular Rheumatism):ti,ab,kw OR (Fibrositis):ti,ab,kw |
| #4 | (Fibrositides):ti,ab,kw OR (Myofascial Pain Syndrome, Diffuse):ti,ab,kw OR (Diffuse Myofascial Pain Syndrome):ti,ab,kw OR (Fibromyositis-Fibromyalgia Syndrome):ti,ab,kw OR (Fibromyositis Fibromyalgia Syndrome):ti,ab,kw |
| #5 | (Fibromyositis-Fibromyalgia Syndromes):ti,ab,kw OR (Syndrome, Fibromyositis-Fibromyalgia):ti,ab,kw OR (Syndromes, Fibromyositis-Fibromyalgia):ti,ab,kw |
| #6 | #1 or #2 or #3 or #4 or #5 |
| #7 | (Exercise):ti,ab,kw OR (Exercises):ti,ab,kw OR (Physical Activity):ti,ab,kw OR (Activities, Physical):ti,ab,kw OR (Activity, Physical):ti,ab,kw |
| #8 | (Physical Activities):ti,ab,kw OR (Walking):ti,ab,kw OR (Jogging):ti,ab,kw OR (Running):ti,ab,kw OR (swimming):ti,ab,kw |
| #9 | (cycling):ti,ab,kw |
| #10 | #7 or #8 or #9 |
| #11 | MeSH descriptor: [Resistance Training] explode all trees |
| #12 | (Resistance Training):ti,ab,kw OR (Training, Resistance):ti,ab,kw OR (Strength Training):ti,ab,kw OR (Training, Strength):ti,ab,kw OR (Weight-Lifting Strengthening Program):ti,ab,kw |
| #13 | (Strengthening Program, Weight-Lifting):ti,ab,kw OR (Strengthening Programs, Weight-Lifting):ti,ab,kw OR (Weight Lifting Strengthening Program):ti,ab,kw OR (Weight-Lifting Strengthening Programs):ti,ab,kw OR (Weight-Lifting Exercise Program):ti,ab,kw |
| #14 | (Exercise Program, Weight-Lifting):ti,ab,kw OR (Exercise Programs, Weight-Lifting):ti,ab,kw OR (Weight Lifting Exercise Program):ti,ab,kw OR (Weight-Lifting Exercise Programs):ti,ab,kw OR (Weight-Bearing Strengthening Program):ti,ab,kw |
| #15 | (Strengthening Program, Weight-Bearing):ti,ab,kw OR (Strengthening Programs, Weight-Bearing):ti,ab,kw OR (Weight-Bearing Exercise Program):ti,ab,kw OR (Exercise Program, Weight-Bearing):ti,ab,kw OR (Weight Bearing Exercise Program):ti,ab,kw |
| #16 | (free weights):ti,ab,kw OR (weight machines):ti,ab,kw OR (elastic bands):ti,ab,kw OR (body weight exercises):ti,ab,kw OR (Weight-Bearing Exercise Programs):ti,ab,kw |
| #17 | #11 or #12 or #13 or #14 or #15 or #16 |
| #18 | (Mind-body exercise):ti,ab,kw OR (physical and cognitive training):ti,ab,kw OR (meditation):ti,ab,kw OR (Relaxations):ti,ab,kw OR (progressive relaxation):ti,ab,kw |
| #19 | (flexibility training):ti,ab,kw OR (balance training):ti,ab,kw OR (yoga):ti,ab,kw OR (Tai ji):ti,ab,kw OR (tai chi):ti,ab,kw |
| #20 | (Taijiquan):ti,ab,kw AND (Qigong):ti,ab,kw AND (Ch'i Kung):ti,ab,kw |
| #21 | #18 OR #19 OR #20 |
| #22 | #10 or #17 or #21 |
| #23 | #6 and #22 |

**3.Embase**

| Search number | Query |
| --- | --- |
| #1 | 'fibromyalgia'/exp |
| #2 | **fibromyalgia**:ab,ti OR **fibromyalgias**:ab,ti OR **'fibromyalgia-fibromyositis syndrome'**:ab,ti OR **'fibromyalgia fibromyositis syndrome'**:ab,ti OR **'syndrome, fibromyalgia-fibromyositis'**:ab,ti OR **'rheumatism, muscular'**:ab,ti OR **'muscular rheumatism'**:ab,ti OR **fibrositis**:ab,ti OR **fibrositides**:ab,ti OR **'myofascial pain syndrome, diffuse'**:ab,ti OR **'diffuse myofascial pain syndrome'**:ab,ti OR **'fibromyositis-fibromyalgia syndrome'**:ab,ti OR **'fibromyositis fibromyalgia syndrome'**:ab,ti OR **'syndrome, fibromyositis-fibromyalgia'**:ab,ti |
| #3 | #1 OR #2 |
| #4 | 'exercise'/exp |
| #5 | exercises:ab,ti OR 'physical activity':ab,ti OR 'activities, physical':ab,ti OR 'activity, physical':ab,ti OR 'physical activities':ab,ti OR walking:ab,ti OR jogging:ab,ti OR swimming:ab,ti OR running:ab,ti OR cycling:ab,ti |
| #6 | #4 OR #5 |
| #7 | 'resistance training'/exp |
| #8 | 'training, resistance':ab,ti OR 'strength training':ab,ti OR 'training, strength':ab,ti OR 'weight-lifting strengthening program':ab,ti OR 'strengthening program, weight-lifting':ab,ti OR 'weight lifting strengthening program':ab,ti OR 'exercise program, weight-lifting':ab,ti OR 'weight-lifting exercise programs':ab,ti OR 'strengthening program, weight-bearing':ab,ti OR 'weight-bearing exercise program':ab,ti OR 'free weights':ab,ti OR 'weight machines':ab,ti OR 'elastic bands':ab,ti OR 'body weight exercises':ab,ti |
| #9 | #7 OR #8 |
| #10 | 'mind-body exercise':ab,ti OR (physical:ab,ti AND 'cognitive training':ab,ti) OR meditation:ab,ti OR relaxations:ab,ti OR 'flexibility training':ab,ti OR 'progressive relaxation':ab,ti OR 'balance training':ab,ti OR yoga:ab,ti OR 'tai ji':ab,ti OR 'tai chi':ab,ti OR taijiquan:ab,ti OR qigong:ab,ti OR 'chi kung':ab,ti |
| #11 | #6 OR #9 OR #10 |
| #12 | #3 AND #11 |
| #13 |  |

**4.Web of science**

| Search number | Query |
| --- | --- |
| #1 | Fibromyalgia (theme) or Fibromyalgias (theme) or **Fibromyalgia-Fibromyositis** **Syndrome** (theme). or Fibromyalgia Fibromyositis Syndrome (theme) or **Syndrome, Fibromyalgia-Fibromyositis** (theme) or **Syndromes, Fibromyalgia-Fibromyositis** (theme) or **Rheumatism,** Muscular (theme) or **Muscular Rheumatism** (theme) or **Fibrositis** (theme) or **Fibrositides** (theme) or **Myofascial** Pain Syndrome, Diffuse (theme) or **Diffuse Myofascial Pain Syndrome** (theme) or **Fibromyositis-Fibromyalgia Syndrome** (theme). |
| #2 | **Exercise** (theme) or **Exercises** (theme) or **Physical Activity** (theme) or **Activities,**Physical (theme) or **Activity,** Physical (theme) or **Physical Activities** (theme) or **Walking** (theme) or **Jogging** (theme) or **Running** (theme) or **swimming** (theme) or **cycling** (theme). |
| #3 | **Resistance Training** (theme) or **Training, Resistance** (theme) or **Strength Training** (theme) or **Training, Strength** (theme) or **Weight-Lifting Strengthening Program** (theme) or **Strengthening Program, Weight-Lifting** (theme) or **Weight Lifting Strengthening Program** (theme) or **Weight-Lifting Exercise Program** (theme) or **Exercise Programs, Weight-Lifting** (theme) or **body weight exercises** (theme) or **free weights** (theme) or **weight machines** (theme) or **elastic bands** (theme) |
| #4 | **Meditation** (theme) or **physical and cognitive training** (theme) or **Mind-body exercise** (theme) or **Relaxations** (theme) or **progressive relaxation** (theme) or **flexibility training** ( Theme) or **balance training** (theme) or **yoga** (theme) or **Tai ji** (theme) or **tai chi** (theme) or **Taijiquan** (theme) or **Qigong** (theme). or **Ch'i Kung** (theme). |
| #5 | **#2 OR #3 OR #4** |
| #6 | **#1 AND #5** |

**Supplementary Table 2. League table regarding FIQ**

| combine_aero_mind | 0.42 (-0.28,1.11) | 0.52 (-0.13,1.17) | -0.11 (-0.63,0.42) | 0.58 (0.01,1.14) | -0.57 (-1.40,0.27) |
| --- | --- | --- | --- | --- | --- |
| -0.42 (-1.11,0.28) | combine | 0.11 (-0.56,0.77) | -0.52 (-1.07,0.02 | 0.16 (-0.42,0.74) | -0.62 (-1.16,-0.08) |
| -0.52 (-1.17,0.13) | -0.11 (-0.77,0.56) | Resistance | -0.63 (-1.12,-0.14) | 0.05 (-0.48,0.59) | -0.39 (-0.69,-0.09) |
| 0.11 (-0.42,0.63) | 0.52 (-0.02,1.07) | 0.63 (0.14,1.12) | Mind_Body | 0.68 (0.32,1.05) | 0.28 (-0.30,0.87) |
| -0.58 (-1.14,-0.01) | -0.16 (-0.74,0.42) | -0.05 (-0.59,0.48) | -0.68 (-1.05,-0.32) | DailyCare | -0.79 (-1.33,-0.25) |
| 0.57 (-0.27,1.40) | 0.62 (0.08,1.16) | 0.39 (0.09,0.69) | -0.28 (-0.87,0.30) | 0.79 (0.25,1.33) | Aerobic |

**Supplementary Table 3. League table regarding VAS**

| combine_aero_mind | 0.86 (-0.04,1.76) | 0.78 (0.02,1.54) | 1.58 (0.49,2.67) | 1.62 (0.90,2.35) | 0.07 (-1.00,1.13) |
| --- | --- | --- | --- | --- | --- |
| -0.86 (-1.76,0.04) | combine | -0.08 (-0.94,0.78) | 0.72 (-0.45,1.88) | 0.76 (-0.07,1.59) | -1.18 (-2.11,-0.24) |
| -0.78 (-1.54,-0.02) | 0.08 (-0.78,0.94) | Resistance | 0.80 (-0.26,1.86) | 0.85 (0.17,1.52) | -1.22 (-1.68,-0.77) |
| -1.58 (-2.67,-0.49) | -0.72 (-1.88,0.45) | -0.80 (-1.86,0.26) | Mind_Body | 0.05 (-0.77,0.86) | 1.38 (0.64,2.12) |
| -1.62 (-2.35,-0.90) | -0.76 (-1.59,0.07) | -0.85 (-1.52,-0.17) | -0.05 (-0.86,0.77) | DailyCare | -0.10 (-0.79,0.59) |
| -0.07 (-1.13,1.00) | 1.18 (0.24,2.11) | 1.22 (0.77,1.68) | -1.38 (-2.12,-0.64) | 0.10 (-0.59,0.79) | Aerobic |

**Supplementary Table 4. League table regarding 6MWT**

| combine | 19.11 (-28.05,66.28) | 22.85 (-14.29,59.98) | -10.18 (-46.23,25.86) | 13.01 (-22.36,48.37) |
| --- | --- | --- | --- | --- |
| -19.11 (-66.28,28.05) | Resistance | 3.74 (-32.30,39.77) | -29.30 (-59.70,1.10) | -6.10 (-40.29,28.08) |
| -22.85 (-59.98,14.29) | -3.74 (-39.77,32.30) | Mind_Body | -33.03 (-52.34,-13.73) | -9.84 (-21.12,1.44) |
| 10.18 (-25.86,46.23) | 29.30 (-1.10,59.70) | 33.03 (13.73,52.34) | DailyCare | 23.19 (7.60,38.78) |
| -13.01 (-48.37,22.36) | 6.10 (-28.08,40.29) | 9.84 (-1.44,21.12) | -23.19 (-38.78,-7.60) | Aerobic |


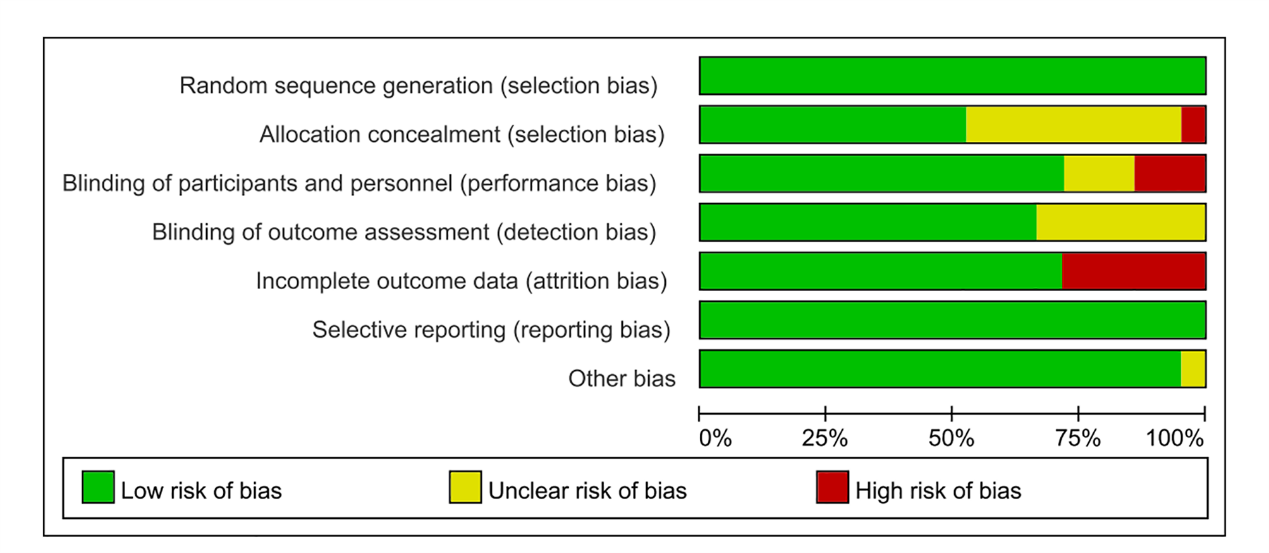


**Supplementary Figure 1.** Risk of bias graph (Percentage form)


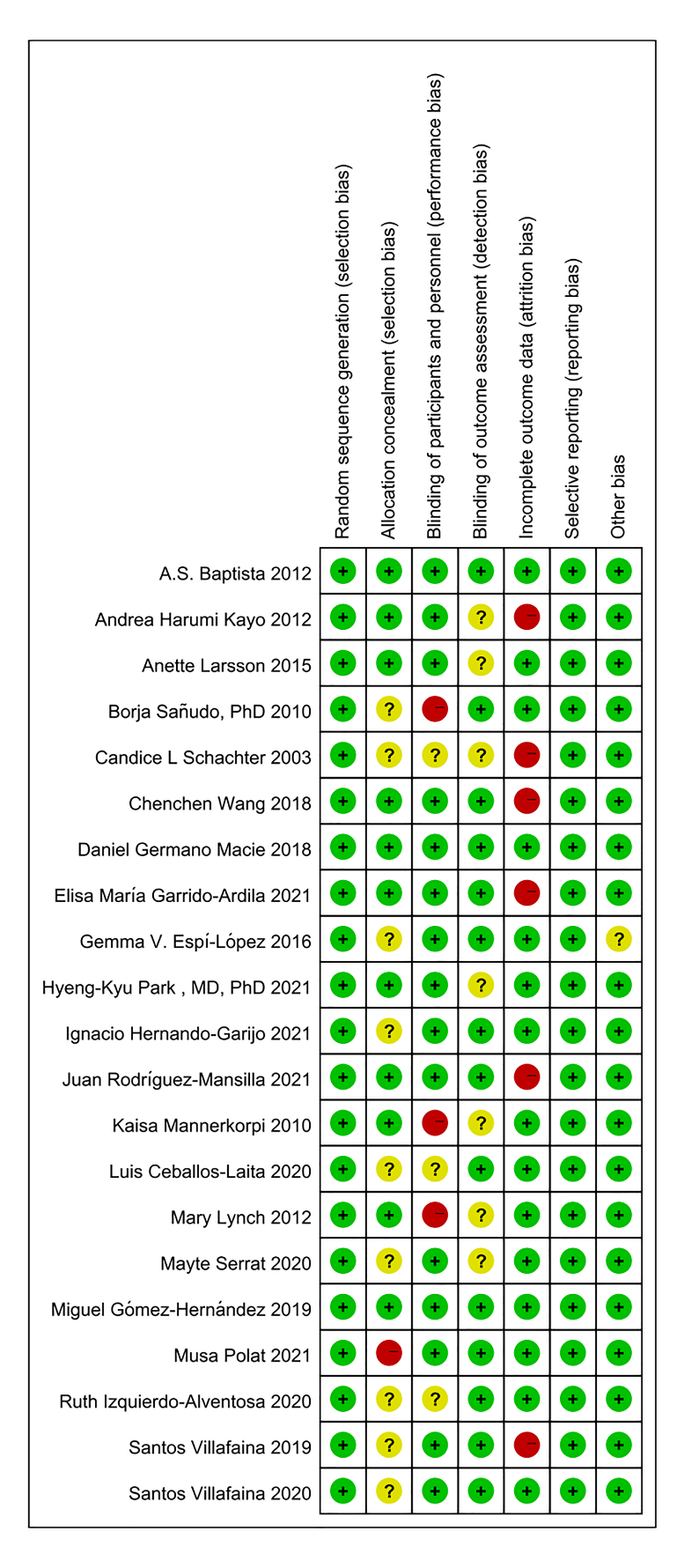


**Supplementary Figure 2**. Risk of bias graph


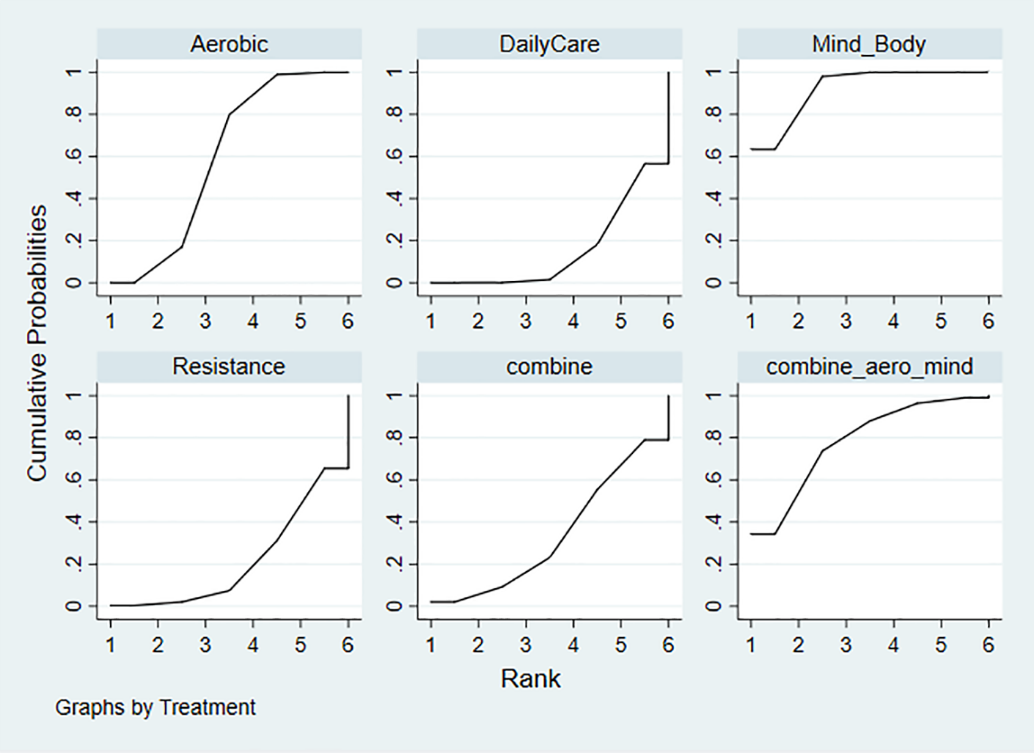


**Supplementary Figure 3.** Meta-analysis of FIQ


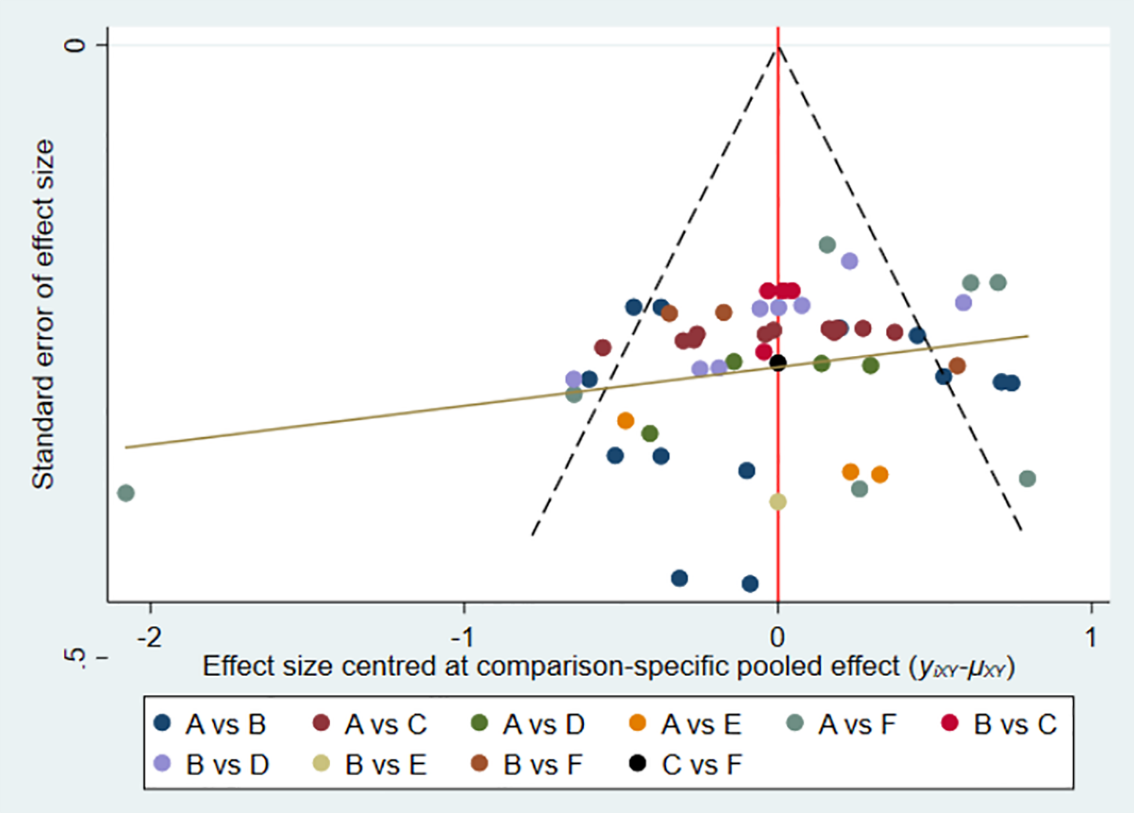


**Supplementary Figure 4.** Funnel plot regarding FIQ


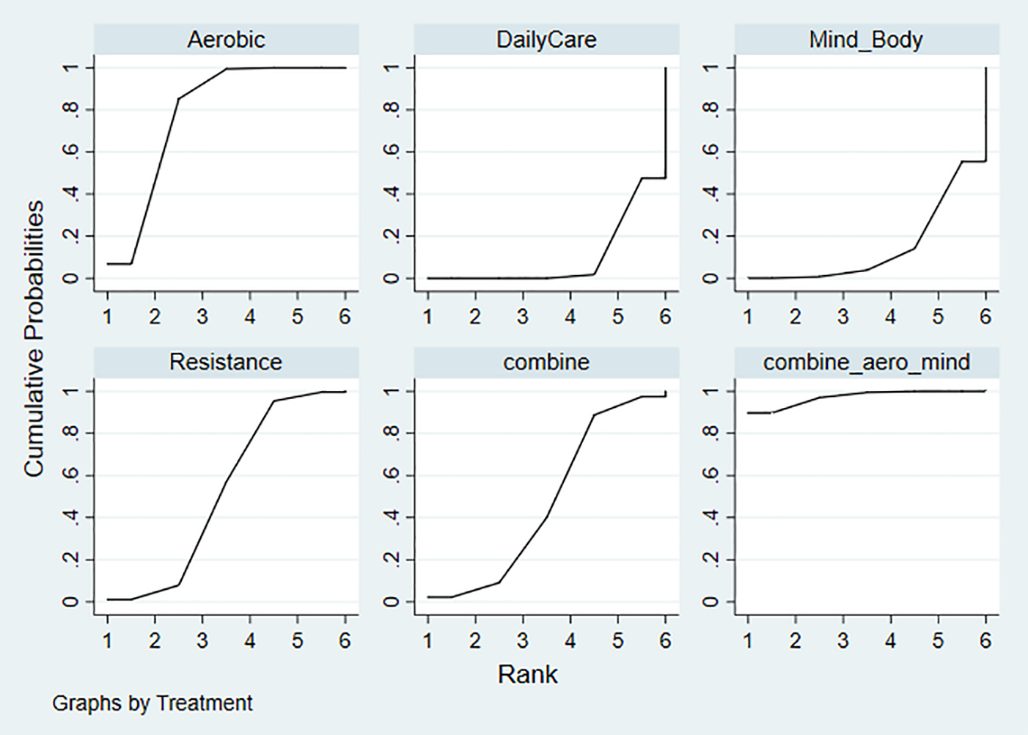


**Supplementary Figure 5.** Meta-analysis of VAS


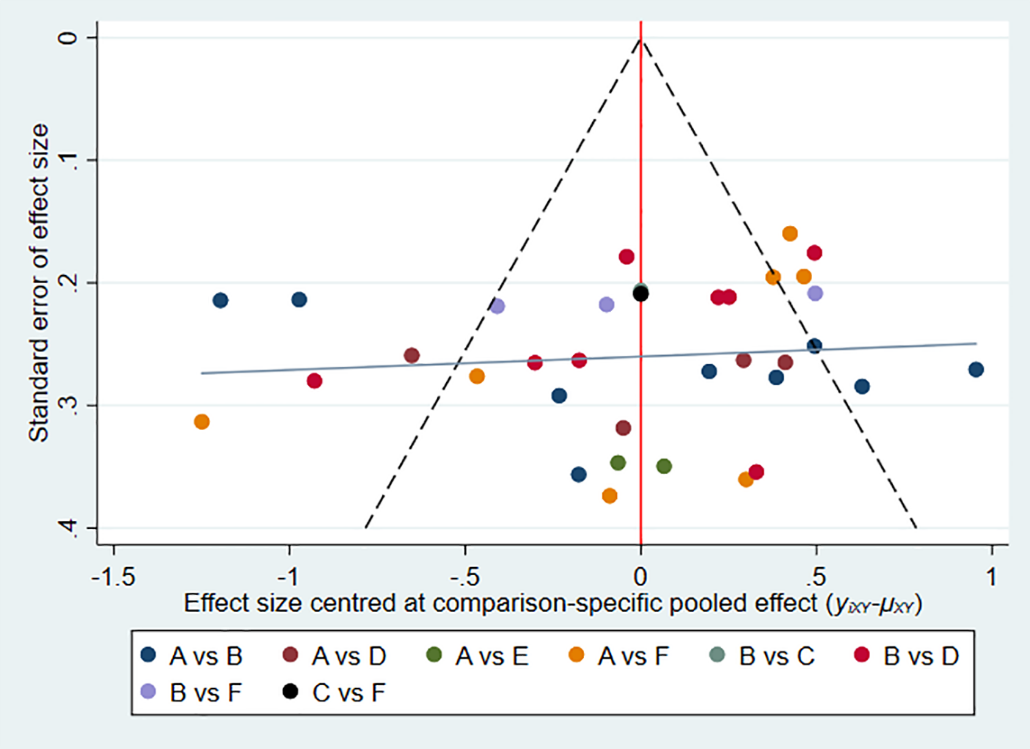


**Supplementary Figure 6.** Funnel plot regarding VAS


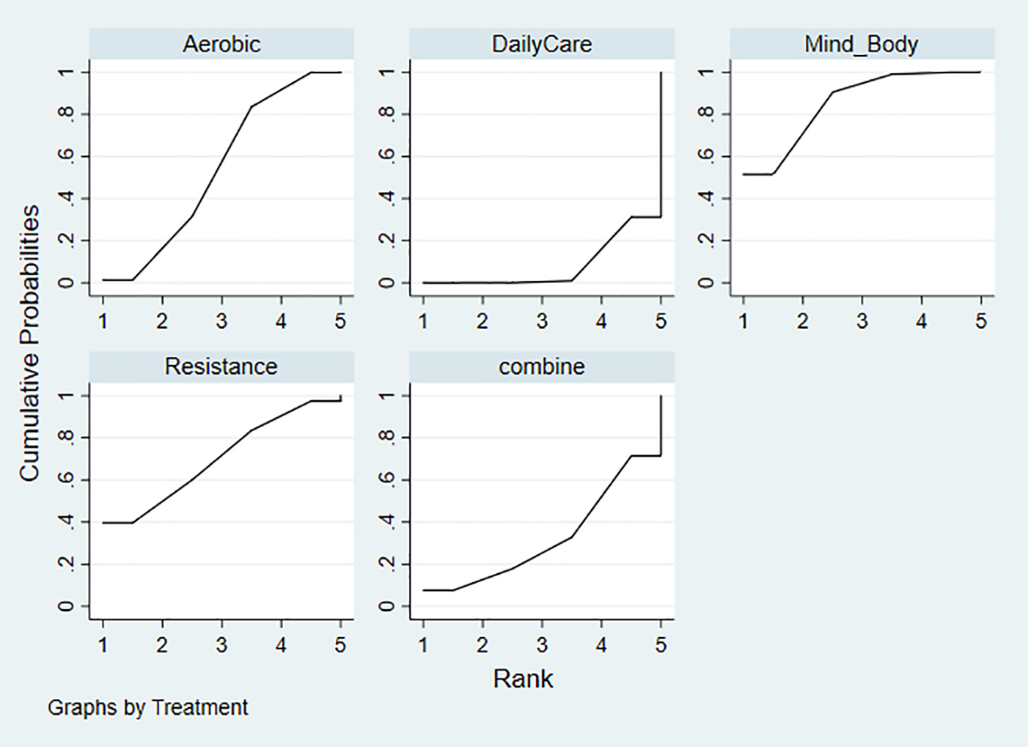


**Supplementary Figure 7.** Meta-analysis of 6MWT


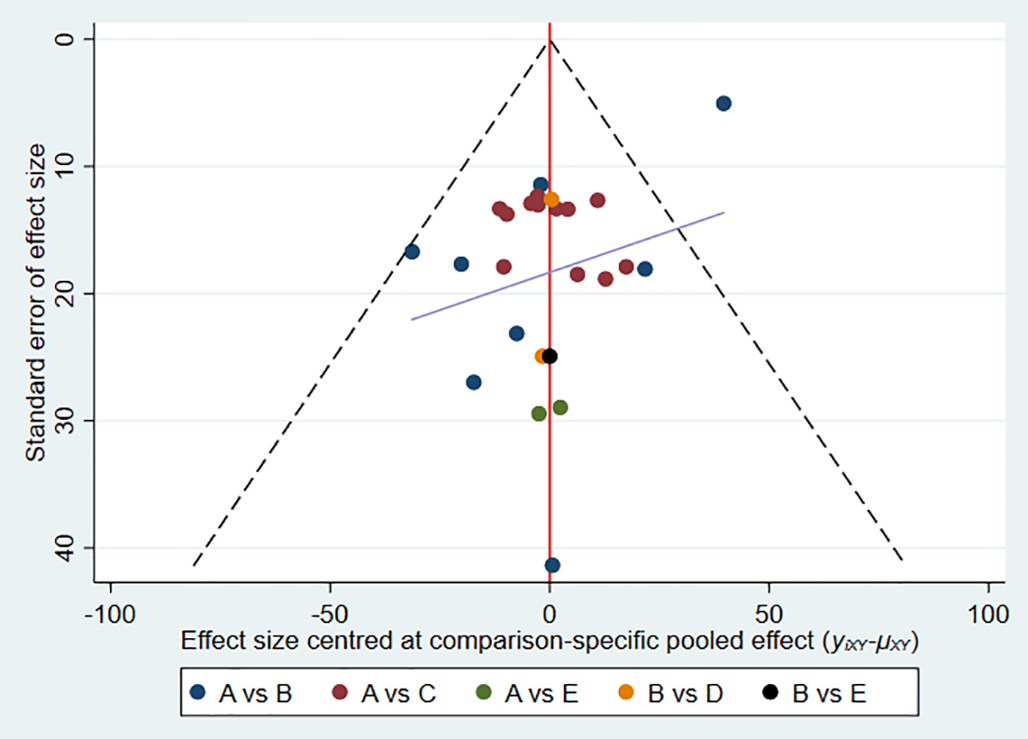


**Supplementary Figure 8.** Funnel plot regarding 6MWT
